# The cervical cancer prevention programme in Costa Rica

**DOI:** 10.3332/ecancer.2015.578

**Published:** 2015-10-08

**Authors:** Ileana Quirós Rojas

**Affiliations:** Coordinación Técnica del Cáncer, Caja Costarricense de Seguro Social, San José, Costa Rica

**Keywords:** cervical cancer, prevention programme, early detection in Costa Rica

## Abstract

Cervical and uterine cancer continues to be an important issue for women around the world, although neoplasia has the greatest demonstrated potential for prevention.

Costa Rica has achieved important advances in the reduction of the incidence and mortality of these cancers since the last century. This is the result of a series of policies, programmes, and plans, not only at the level of the health care system, but also in other areas.

Increased access for women to care in health centres, fundamentally at the primary level, has been vital, as has ensuring the quality of cytology readings and access to diagnosis and treatment for precursor lesions for *in situ* and invasive cancers.

Despite all of these achievements, there are still challenges to be overcome, which are widespread in many countries in Latin America and the Caribbean.

It is important to learn from the experiences of other countries in order to improve women’s health not only as a health objective, but also as an ethical imperative to promote the exercise of women’s rights to life and health.

## Background

Cancer has been one of the concerns of the health authorities in Costa Rica since the beginning of the last century. In 1940, one of the first laws on the subject was passed: the development of a Cancer Institute, which did not become a reality as said law was repealed and gave rise in 1949 to the creation of the Department to Fight Cancer in the Ministry of Health at that time [1].

These actions occurred within the context of very important social changes for the country: the social reforms, such as the creation of the Costa Rican Social Insurance Agency (C.C.S.S.), financed in a tripartite manner by the insured, the employer, and the state, the labour code, the environmental health programmes, and nutrition and vaccination programmes, among others. These policies made it possible to improve the living conditions of the population through their redistributive effects.

In the 1970s, the programmes to extend both the vertical and the horizontal coverage of social insurance to reach the goal of universal coverage were intensified. These actions reinforced the infrastructure, the training of professionals and technicians, and the provision of medicines and equipment for the prevention and treatment of the conditions prevalent at the time [2]. In 1973, the general health law was created, which established health as a benefit in the public interest under the protection of the state [3].

At the same time, a series of programmes of primary care were consolidated, such as the rural and community health programme, which brought preventive care to the farthest corners of the country and achieved a great impact on the health of the population, and consequently improved the demographic health indicators in Costa Rica. In the same manner, the records of vital statistics were greatly improved and the National Tumour Registry was created in 1976 [4].

Within the framework of these reforms, in 1960, the national plan for the detection of cervical cancer was established. Detection by means of cervico vaginal cytology testing was included in the so-called ‘family planning visits’ both with the social insurance and the ministry of health.

A significant decrease in the number of deaths due to cervical cancer began in the mid-1960s. From 1965 to 1980, the mortality rates were reduced to less than half [5, 6] and invasive cancer rates declined from 50/100,000 women to 36/100,000 women [4, 5]. This figure is considered reliable due to the quality of the data of the national tumour registry and vital statistics. Likewise, the reduction in deaths and cases of invasive cancer occurred in all of the age groups, achieving an inversion of the *in situ*/invasive ratio.

In the 1980s and 1990s, two reforms in the health sector were implemented: the first consisted of the integration of the services of the Costa Rican Social Insurance Agency, which were mainly curative, and those of the Ministry of Health, which were preventive, and the second and more importantis the transfer of services for direct patient care to the people and programmes of the C.C.S.S. and the strengthening of the governing role of the Ministry of Health. As part of this last reform, the health areas were created with the Basic Integrated Care Teams (EBAIS) which made up the primary care level and their progressive implementation substantially improved access to health services throughout the country.

A study by Rosero demonstrated the following result: ‘the percentage of persons without equitable access to primary health care services was reduced by 15% between 1994 and 2000 in the areas which adopted the reform in 1995–1996, while in the areas where the reform had not been adopted in 2000, the reduction was of only 3%’ [7].

**Figure 1.**
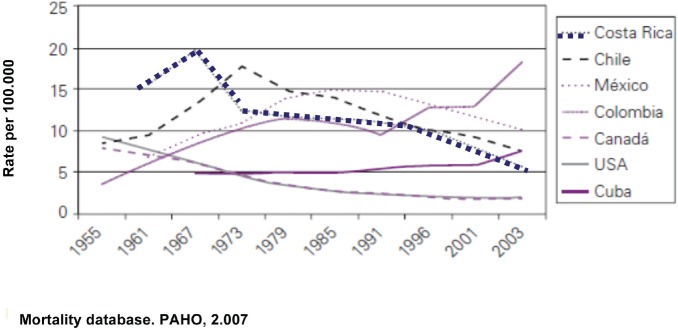
Trends in the mortality rates due to cervico-uterine cancer standardized for age: selected countries, 1955–2003

**Source:** Pan-American Health Organization Regional Strategy and Action Plan for the prevention and control of cervico-uterine cancer in Latin America and the Caribbean, p. 11.

The trend toward a decrease in mortality is shown in Figure 1, where the changes mentioned coincide with the policy changes described in the different periods.

The results described reflect the improvement in living conditions, fundamentally access to education and health services along with specific policies and programmes which likely favoured the conditions for reducing the incidence of and mortality from cervicouterine cancer and improve the quality of life of women and their families. This is a result of the fact that this is the cancer that causes the highest number of premature deaths among women and thus the greatest loss of healthy and productive years of life.

A National University study calculated that the economic value of unpaid housework done by women in Costa Rican homes represents 16% of the gross domestic product (GDP) in the case of the greater metropolitan area (GMA), which reinforces the impact that this loss of women’s lives has on the economy of the country [8].

## Current situation of cervical cancer in Costa Rica

**Figure 2.**
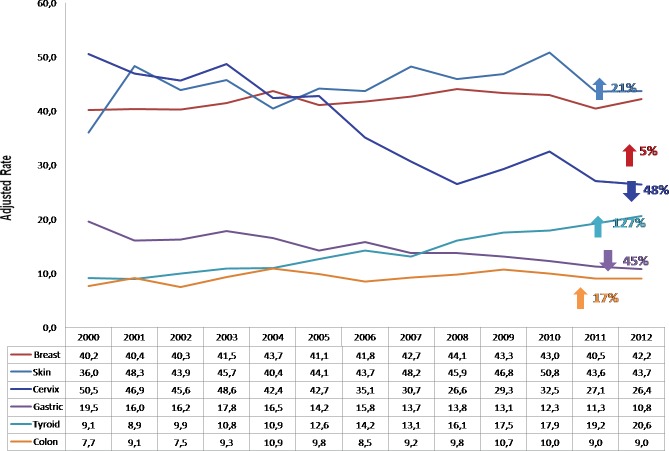
Incidence of most common malignant tumours in women by year, Costa Rica 2000–2012 (rate adjusted per 100,000 women).

**Source:** National Tumor Registry, 2.014.

**Figure 3.**
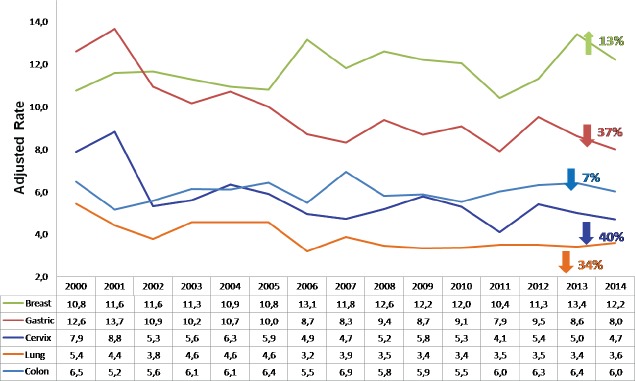
Mortality due to most common malignant tumours in women by year Costa Rica 2000–2013 (rate adjusted per 100,000 women).

**Source:** National Tumor Registry, 2.014.

In Costa Rica, this cancer continues to be one of the most common neoplasias in the female population: it has the third highest incidence and the fourth highest mortality due to cancer despite the important reduction, which has been achieved in both rates. The incidence was reduced by 1.85 times between 2000 and 2011, and the mortality by 1.58 times between 2000 and 2013 (Figures 2 and 3).

**Figure 4.**
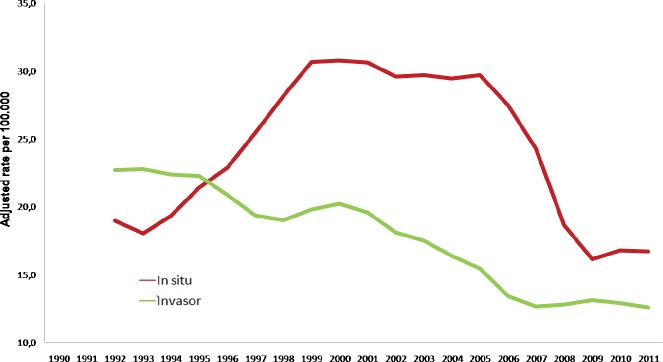
Trends in the incidence of *in situ* and invasive cervical cancer, Costa Rica, 1990–2011. (Rate adjusted per 100,000 women, moving average for 3 periods).

**Source:** Project for Improving Integrated Care for Cancer, C.C.S.S. prepared based on data from the National Tumour Registry, 2014.

The ratio of invasive to *in situ* cancers was inverted as of 1995 (Figure 4).

**Figure 5.**
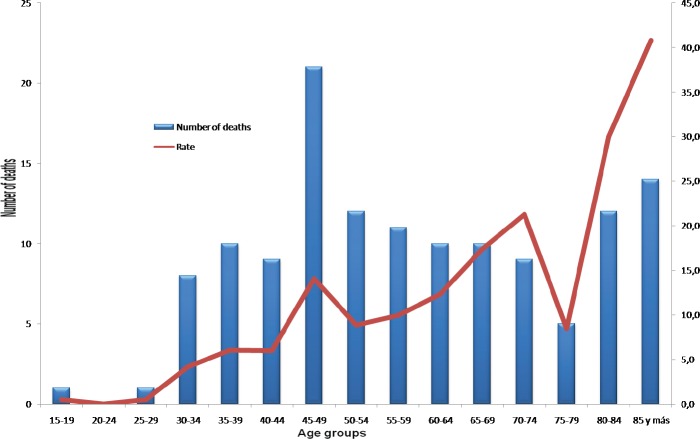
Deaths and specific mortality rates by age for cervical cancer by five-year age groups, Costa Rica, 2013 (rate per 100,000 women).

**Source:** Project for Improving Integrated Care for Cancer, C.C.S.S. prepared based on data from the National Tumour Registry, 2014.

In general, the majority of the deaths due to this cause occur in middle-aged women (between 45 and 64 years old), thus accumulating a larger number of years of life lost (Figure 5).

**Figure 6.**
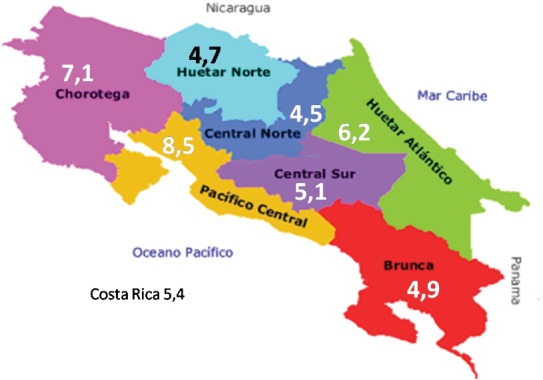
Five-year mortality rates due to cervical cancer by region, Costa Rica, 2007–2011. (gross rate per 100,000 women).

**Source:** Project for Improving Integrated Care for Cancer, C.C.S.S. prepared based on data from the National Tumour Registry, 2014.

Although global rates have been reduced, there is a different pattern in the seven provinces. In Table 1, the disparities among the same are evident. While in Cartago, for each 3.6 cases of cancer *in situ* diagnosed, one invasive case is diagnosed, in Guanacaste, the rate is one to one, and in Limón, it is inverted, with 1.6 cases of invasive cancer diagnosed for each case of cancer *in situ*. As can be observed on the map (Figure 6), the coastal regions are most affected, as they correspond to the provinces with the highest number of deaths due to this neoplasia.

A study of survival with cervical cancer confirms the impact of the reduction of the same. This study included a cohort of patients diagnosed in 1999, and it was observed that the accumulated probability of survival at 5 years for the entire population was 88.3%. The women whose cancers were detected *in situ* at the time of diagnosis showed an accumulated survival probability of 98.8%, while those with a diagnosis of invasive carcinoma showed a survival rate of 68.3%. This difference was statistically significant.

The authors concluded that the results obtained showed numbers that were higher than those found in other countries with a lower level of development than Costa Rica and similar to those found in developed nations. With the results obtained, one can conclude that the strategies and actions implemented to achieve the reductions found in the incidence and mortality were effective and sustainable through time [9].

Although progress in the prevention and treatment of cervical cancer is evident, the incidence of invasive cervical cancer shows a very slow reduction, which implies that detection is still occurring late.

## Programme for the prevention and treatment of cervical cancer

The consolidation of a series of interventions for the prevention and treatment of cervical cancer in the 1980s and 1990s achieved a rate of between 70 and 74% of women of fertile age had at least one cytology test during their lifetime, as indicated by the Health and Fertility Surveys and Reproductive Health, Fertility, and Family Structure Survey (EFES-86 and ENSR FFF 93) [5, 6].

During the 1990s, the technical authorities of the C.C.S.S. and the Ministry of Health conducted an evaluation of the problem of cervical cancer and offered proposals to improve the response of the health care system in terms of prevention: the centralisation of cytology readings in a single centre of the C.C.S.S., as well as improvements to the diagnostic and treatment services for precancerous lesions.

In 1998, the ‘National Programme for the Prevention and Integrated Treatment of Cervical and Breast Cancer’ was launched by the Social Insurance Authority. Thus, the National Cytology Centre established national norms for the screening, diagnosis, and treatment of precursor lesions to cervical cancer in women between 20 and 64 years of age with the creation of: ‘The Manual of Standards and Procedures for Integrated Care for Women for the Prevention and Management of Cervical Cancer’, I and II ‘Level of Care and Standards for Cytology Labs’, in effect from 2007 to the present and the provision of equipment to meet these standards [1]. These changes occurred concomitantly with the implementation of the Health Sector Reform, in which the C.C.S.S. took over the content and actions of the Cancer Programme of the Health Ministry, which transferred the services of direct care to the same and assumed its governing and regulating role in the area of health monitoring and national standards.

This is how the C.C.S.S. came to provide these services throughout the country to those who require them. The interventions and strategies of the programme are established by the technical standards authority of the central level of the Institution and articulated with the other basic interventions defined for each age group within the various areas of practice of the health teams.

The supplies for screening and treatment are purchased in a centralised manner, as are all of the other supplies including medicines, with the institutional budget financed by the health insurance programme. Each centre plans and uses its assigned budget in order to fulfil the goals established at the institutional level within the framework of the current health policies.

The evaluation of the actions and goals is done by means of monitoring the data on incidence and mortality from the National Tumour Registry (RNT) and the National Institute of Statistics and Censuses (INEC), the performance indicators for the services at the primary and specialised levels. The latter are agreed upon previously with the Health Services Purchasing unit of the Institution. Likewise, the results of the cytology centre are monitored, and the centre also belongs to the C.C.S.S., as mentioned previously.

## The cervical cancer care plan

As in most of the countries of the world, the governments demonstrated their concern with the magnitude of the problem of cancer. In 2009, the Board of Directors of the C.C.S.S. declared cancer a topic of interest and institutional priority. In this way, they seek to prioritise actions and investment in the resources to improve the prevention and care of people at risk or with any of the cancers that have been determined to be of priority, among them cervical cancer.

In 2012, under the framework of the National Cancer Plan (PNC), coordinated by the Ministry of Health, an Institutional Plan for Cancer Care (PIAC) 2012–2016 was established, which combined prior efforts and articulated and instrumented a series of actions to put the institutional and governmental mandate into action [10, 11].

Thus, it was decided to implement a care plan which permitted intervention in the weak areas to allow for improvement in the effectiveness of the programme.

Within this context, an analysis was performed to determine the strengths and weaknesses.

Costa Rica possesses a series of strengths that can facilitate the reduction of the cases and deaths from this cancer, as shown at the beginning of this study [11]:

- A service network with national coverage
- Access for the majority of women to both public and private health services
- A centralised laboratory that meets the quality standards
- The goals for access and continuity of the interventions for prevention and treatment in this area are incorporated in the health care services purchasing plan, the operational plans, and the budget as well as in the evaluation of the performance of the health services.
- A system for the transportation, receipt, processing, reading, reporting, and return of results of cytology testing to the users within a period of no more than 8 days.
- Medium and high-complexity centres (clinics and hospitals) which have the resources to care for women who have precursor lesions or cervical cancer and which are treating the women referred to them within the time frames established for each of the conditions, which ensures continuity of care. The women with cytology results of malignant *in situ* or invasive lesions are treated within the 8 days following the referral in nearly 100% of cases treated in hospitals.
- An institutional system for the logistics of the acquisition of supplies to ensure the permanent availability of medications, instruments, teams and other needs for screening and treatment.

Among the aspects in need of improvement are as follows: [11]

- The current national standard has not been updated in terms of the age for the start of screening or the technology to complete it.
- Administrative barriers: Most women must request a doctor’s appointment in order to have the cytology test done, and in some centres, opportunities are lost.
- The information and counselling in sexual and reproductive health are not integrated in a generalised manner with the health services in the different scenarios of work with men and women of different ages and stages of life.
- Young women are given priority for cytology testing.
- Inadequate application of the existing treatment standards.
- Active searches of the target population for screening in all health areas are not done.
- Diverse criteria are applied at the first level for testing, interpretation, and reference of women with altered cytology results due to a lack of knowledge and supervision for compliance with the standards.
- The response of the colposcopy services is sufficient, but some centres exceed the established time period to treat a patient with a precursor lesion.
- Insufficient information for women relating to the different aspects of screening as well as the resources and procedures available for confirmation under the different regimes that exist.
- Underutilisation of human resources, fundamentally of nursing staff by the health areas, who could make screening more accessible.
- Disarticulation of the sources of information that makes it difficult to determine actual coverage and calculate the indicators, which permit the monitoring and evaluation of different interventions of the programme.
- The absence of a nominal registry at the national monitoring level to confirm coverage by the public and private sector, as the only registries are those of the institutional data collected by the CCSS.

These weaknesses point to three critical areas: local management, access to and control of information on the prevention of cervical cancer by women, the application of the standards on the part of health care personnel, and the information system.

To meet these challenges, strategies are being developed with actions that permit advancement of the articulation and reinforcement of a national programme, which coordinates and manages the actions at the various levels.

The important action are as follows: updating the national standards in conjunction with the Ministry of Health, incorporating HPV and new handling protocols into DNA testing, evaluation of the incorporation of the HPV vaccine into the basic national scheme, as well as supporting regional management in putting these into operation. All of this is in order to improve the effectiveness of the programme.

The assessment of the experience of the Guanacaste Epidemiological Project is a strategic method for analysing and applying the good practices developed by the same.

The most important challenge is improving the management of the services so that integrated, whole care services can be provided to women, as the set of problems derives from the structure and organisational culture of the services.

Also included among the challenges is monitoring of the programme indicators at the national level which includes data from both the public and private sectors. This is because the institutional Papanicolau coverage does not reflect the national level, as many women receive care in the private sector, and these tests are not analysed at the cytology centre.

This is very important, as the C.C.S.S. must multiply its efforts to reach the women who do not have access to these interventions, and are those who have a greater risk and are probably living in conditions of social vulnerability.

## Conclusions

Reducing this type of cancer depends in large part on the social response of the health care services. The experience of Costa Rica demonstrates that the social policies maintained for decades have created suitable conditions for a national programme of cervical cancer prevention, detection, diagnosis, and treatment to reach its goals.

The investment in the development of human capital, the infrastructure and equipment, the implementation of a set of interventions intended to improve the quality of care for patients, particularly women, uniting the committed efforts of all of the health care teams in the country, these have been the key aspects of achieving the indicators that exist now.

Nevertheless, reality is always changing and becoming more complicated, as new problems and critical issues are always being presented. Thus, it is necessary to maintain active vigilance, proposing strategic actions which allow for overcoming weaknesses and reducing risks.

This is the greatest challenge which confronts our health care system. And this is why we must continue our efforts directed at articulating and reinforcing a national programme which ensures the coverage, quality, and effectiveness of the interventions.

Achieving these goals will also require a robust information system, appropriate management of network services, and reinforcement of integrated and whole health care for patients at all stages of life.

The lessons learned and the application of demonstrated good practices both at the internal level in the Guanacaste Epidemiological Project, and in experiences from other countries, are important resources for improving the proposals that are being implemented.

All of this is in order to improve the quality of life of women and society as a whole by reducing unjust and avoidable differences.

## Conflicts of interest

The author declares that no conflict of interest exists.

## Figures and Tables

**Table 1.** Incidence of cervical cancer by performance, according to province. Costa Rica 2010. (Rate adjusted per 100,000 women).

Province	*In situ*		Invasor	
	N°	Rate	N°	Rate
**Costa Rica**	526	20,65	290	11,85
San José	171	18,22	93	9,97
Alajuela	65	13,63	54	11,67
Cartago	122	43,37	34	12,38
Heredia	23	8,81	17	7,15
Guanacaste	39	26,48	39	25,90
Puntarenas	50	25,85	19	10,39
Limón	21	10,59	34	17,27
Ignored^*^	35		0	

* 35 cases are included in which residence was not reported.

**Source:** Health Monitoring Directorate. National Tumour Registry.
